# The optimization of college tennis training and teaching under deep learning

**DOI:** 10.1016/j.heliyon.2024.e25954

**Published:** 2024-02-11

**Authors:** Yu Zhang

**Affiliations:** Department of Social Sciences, Zhejiang College of Security Technology, Wenzhou, 325016, China

**Keywords:** Physical education teaching evaluation, Deep learning, BPNN, Tennis tactics, Diagnostic model

## Abstract

To enhance the integration of deep learning into tennis education and instigate reforms in sports programs, this paper employs deep learning techniques to analyze tennis tactics. The experiments initially introduce the concepts of sports science and backpropagation neural networks. Subsequently, these theories are applied to formulate a comprehensive system of tennis tactical diagnostic indicators, encompassing construction principles, basic requirements, diagnostic indicator content, and evaluation indicator design. Simultaneously, a Back Propagation Neural Network (BPNN) is utilized to construct a tennis tactical diagnostic model. The paper concludes with a series of experiments conducted to validate the effectiveness of the constructed indicator system and diagnostic model. The results indicate the excellent performance of the neural network model when trained on tennis match data, with a mean squared error of 0.00037146 on the validation set and 0.0104 on the training set. This demonstrates the outstanding predictive capability of the model. Additionally, the system proves capable of providing detailed tactical application analysis when employing the tennis tactical diagnostic indicator system for real-time athlete diagnosis. This functionality offers robust support for effective training and coaching during matches. In summary, this paper aims to evaluate athletes' performance by constructing a diagnostic system, providing a solid reference for optimizing tennis training and education. The insights offered by this paper have the potential to drive reforms in sports programs, particularly in the realm of tennis education.

## 1 Introduction

### 1.1 Research background and motivations

Competitive tennis is a sport whose primary goal is to win. The competitiveness displayed by athletes in the competition is reflected in five key aspects: physical fitness, intelligence, mental ability, skills, and tactical ability. Tennis belongs to the skill-dominant group of confrontational sports involving rackets and nets. Although physical and mental skills have an increasing impact on athletes' performance in modern tennis, tactical abilities still play a leading role. In a match between high-level athletes, the outcome of every point depends on the quality of each shot and the strategic use of technical and tactical strategies. Moreover, the tactical ability of tennis athletes is a crucial factor in determining their competitive potential. The successful implementation of techniques and tactics in a match is a decisive factor in the final result. Therefore, studying the techniques and tactics used in competitive tennis matches can provide a scientific basis for developing athletes' tactical training and decision-making abilities. It is also a meaningful way to improve tennis competition in China. Additionally, there are several issues with the training and teaching of college students in tennis. This paper aims to inspire the further optimization of college tennis teaching and training by diagnosing and evaluating the tactics employed by tennis athletes.

However, individual differences and physiological changes are common in collegiate tennis training. Each student has varying levels of physical fitness, technical proficiency, and physical condition, which limits the effectiveness of traditional one-size-fits-all teaching methods. Deep learning technology can offer personalized training recommendations and feedback to each athlete based on their individual data. By analyzing athletes' physiological parameters, technical movements, and tactical choices, deep learning can identify potential areas of concern and assist coaches in adjusting training plans more effectively. Furthermore, deep learning can be used for risk assessment of sports injuries, enabling early prevention and reduction of injury risks. This introduces an intelligent approach to collegiate tennis training that can enhance training efficiency, minimize injury risks, and better cater to the individual needs of students. This is a promising field that deserves further exploration.

### 1.2 Research objectives

The primary objectives of this paper are to construct a set of tactical diagnostic indicators for men's singles tennis matches and develop a tactical diagnostic model based on the Back Propagation Neural Network (BPNN). The aim is to provide a more scientific method for assessing and diagnosing the tactical performance of athletes in matches, specifically Chinese male tennis players. The main issues can be summarized as follows: how to establish a set of tactical diagnostic indicators suitable for men's singles tennis matches, how to enhance the technical and tactical skills of Chinese tennis players to promote the development of tennis in China, and how to use BPNN to construct a tactical diagnostic model that can evaluate the tactical performance of exceptional male Chinese tennis players and improve their technical and tactical skills.

Specifically:

(1) The first objective is to build a tactical diagnostic index system for men's singles tennis matches. Tactical analysis and diagnostic index systems suitable for tennis are constructed based on previous research, and evaluation standards are determined. This provides researchers with a new method for tactical analysis and diagnosis, thereby enriching the existing methods for analyzing tennis events.
(2) The tactical diagnostic model is constructed using BPNN. Subsequently, the tactical analysis and diagnosis of exceptional Chinese male tennis athletes are conducted using the constructed tactical diagnostic index system and its evaluation criteria. This provides data support for Chinese tennis athletes and helps in improving their technical and tactical abilities. A comprehensive evaluation of the tactical level of outstanding Chinese and foreign athletes can enrich the methods for analyzing men's singles tennis matches and inspire improvements in tennis training and teaching.

## 2 Literature review

There is a significant amount of literature on tennis from the perspective of physiology in foreign countries. For example, the application of cognitive skills and perceptual action training in tennis training is studied from the perspective of sports psychology [1]. A physiological perspective is used to quantify the effect of fatigue on tennis athletes' performance, highlighting the limitations of traditional measurement methods [2]. From the perspective of biomechanics, the mechanical characteristics of the shoulder joint of elite tennis athletes are analyzed during their serve [3]. These results have played an essential role in promoting tennis development, research, and practice in China. Additionally, some scholars have studied the reform of physical education from a medical care perspective. Wei et al. [4] pointed out that public physical education courses in higher vocational colleges face issues such as outdated teaching materials, weak teachers, outdated teaching methods, inadequate course selection management, and low subject status of public physical education courses in higher vocational colleges [4]. Lin [5] highlighted the current situation of physical education teaching in higher vocational colleges. The guiding ideology of school physical education has long been limited to merely enhancing students' physical fitness. The structure of physical education courses is too simplistic, and physical education teaching lacks hierarchy and is overly traditional [5]. Liu and Zhao [6] identified problems with physical education teaching in higher vocational schools, including a narrow concept of education, outdated teaching methods, repetitive teaching content, insufficient teaching hours, and ineffective evaluation of students' physical exercise effects on physical education [6]. The existing research results indicate that the reform of higher vocational physical education is imperative, and the reform of teaching content will be a crucial breakthrough. Jain et al. [7] explored the use of data mining techniques for predicting sports match outcomes and compared them to benchmark models. Through data analysis and mining, this paper aims to provide a more accurate method for predicting sports match results [7]. Jain et al. [8] investigated how to use customer-generated feedback data for airline recommendations. This paper leverages customer feedback data to develop predictive models that offer airlines more personalized services and recommendations. Additionally, it provides a valuable reference for this paper, which focuses on predicting event outcomes based on match data and sports tactics [8].

Tactical diagnosis and analysis work aims to identify statistical indicators and collect data [9]. Currently, the establishment of statistical indicators for tennis tactical diagnosis and analysis research includes self-designed statistical indicators based on research needs and the selection of technical statistical indicators published on the event's official website [10]. Firstly, statistical indicators for the technical effect of the return service are designed, including “score, initiative, average, and turnover” [11]. These indicators are used to analyze the technical impact of serving by the top eight athletes in women's singles at the seventh National Games of China, considering the outcome and result of the game. Subsequently, the “serving success rate” and “serving score” are proposed as analysis indicators for the technical effect of serving [12]. These indicators have been applied to compare the technical movements of excellent Chinese athletes and world-class athletes and analyze the differences. Then, indicators such as the success rate of the first serve are proposed to evaluate the technical and tactical level of athletes' serving [13]. Finally, the 2006 Australian Open women's doubles final is used to diagnose and analyze the winning factors, technical issues, and tactical problems of Chinese athletes. Targeted training suggestions are provided in response to these issues [14].

In terms of DL evaluation, recent empirical research results have shown the importance of DL evaluation methods. DL is increasingly focused on process evaluation and performance evaluation. The assessment mainly includes basic knowledge points, content knowledge, and high-level understanding to cultivate students' influence and emotional ability. Jungo et al. [15] emphasized the interconnectedness and practical application of interdisciplinary knowledge. They effectively deepened students' understanding mechanisms by using relevant negative and positive examples [15]. Hou et al. [16] pointed out the teacher-student evaluation in preschool education. They suggested that this approach could encourage students to internally and critically evaluate themselves and their peers, effectively improving academic performance and students' critical thinking [16].

Through the research literature, there have been many studies on the optimization of college sports training and teaching at this stage. In addition, scholars mainly focus their research on the teaching design of table tennis, basketball, and other aspects. However, tennis is also a popular sports event, and there has been little research done on its teaching. Therefore, this paper mainly focuses on studying the optimization design of tennis teaching for college students. The innovation lies in the introduction of NN technology in DL during the research process, and the completion of the model construction. Additionally, a comprehensive and objective tactical diagnostic index system for men's singles matches in hard court tennis is constructed here. The diagnostic criteria are established to enable a multi-angle, comprehensive, and detailed evaluation of the players' tactical level. The aim of this paper is to enhance the level and effectiveness of tennis teaching by constructing a perfect tennis teaching evaluation system.

## 3 Research methodology

### 3.1 Healthcare and sports

#### 3.1.1 Healthy sport

Healthy sport is an emerging topic that combines the health and sports industries. Sports embody the Olympic spirit with its focus on “higher, faster, and stronger" performance. Health, on the other hand, emphasizes the additional benefits of sports on physical and mental well-being, and downplays the competitive aspect. The concept of healthy sport deserves to be promoted and popularized in national sports [17].

#### 3.1.2 Health care and sports

Healthcare refers to comprehensive measures taken to protect and enhance human health, and prevent diseases. Healthcare sports are activities that aim to rejuvenate the mind and body, and improve overall bodily function by learning and mastering the fundamental theories, knowledge, methods, and skills of physical conditioning. Occupational health sports, on the other hand, focus on learning and mastering theoretical knowledge and skills related to sports, in order to protect and enhance the physical health of practitioners and prevent diseases. The core of physical health education for healthcare purposes is to help people understand the fundamental principles of improving physical fitness and health, and to acquire the relevant knowledge and skills. People should consciously apply these theories in practice to promote self-care. The curriculum for occupational health sports design in medical and health vocational colleges includes both traditional and medical health sports.

#### 3.1.3 Healthcare sports content

Medical sports, also known as physical therapy, are an effective method for preventing, treating, and rehabilitating diseases. The target of medical sports is the patient, and the means is physical exercise. When designing the content of medical and healthcare physical education in physical education teaching at medical and health vocational colleges, the medical professional knowledge mastered by medical and health students should be combined to select, identify, and apply healthcare physical education content. This can improve the professional fitness level and healthcare sports ability of medical and health students, enabling them to effectively guide patients in future professional activities. The physical educationcurriculum for medical and health sports can include compulsory and elective courses such as medical and health sports theory, medical gymnastics, and health massage.

In conclusion, the concept of sports for health contributes to the development of students' physical fitness, while emphasizing the positive impact of sports activities on their physical and mental well-being beyond competition. This approach not only equips students with athletic prowess but also allows them to derive enjoyment from sports.

### 3.2 BPNN

#### 3.2.1 The structure of BPNN

**Fig. 1:**
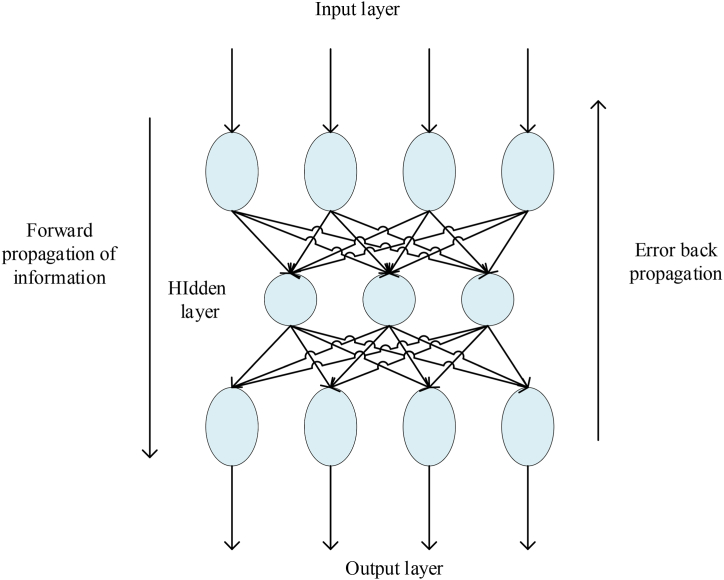
Topological structure of three-layer BPNN.

BPNN is a multilayer feedforward NN. It is characterized by signal forward propagation and error backpropagation [18]. Generally speaking, a typical BPNN has a structure of three or more layers, one input layer, one or more hidden layers, and one output layer, as shown in Fig. 1.

Fig. 1 shows that BPNN (Backpropagation Neural Network) consists of two processes: forward propagation and error backpropagation. In the forward propagation process, the data flows from the input layer to the hidden layer and then to the output layer. When the output layer fails to produce the desired output, the weights are adjusted through reverse error propagation. These two processes alternate, implementing a gradient descent strategy to minimize the error function in the weight vector space. Through dynamic iteration, a set of weight vectors is searched to minimize the network error function [19].

#### 3.2.2 Modeling process of BPNN

**Fig. 2:**
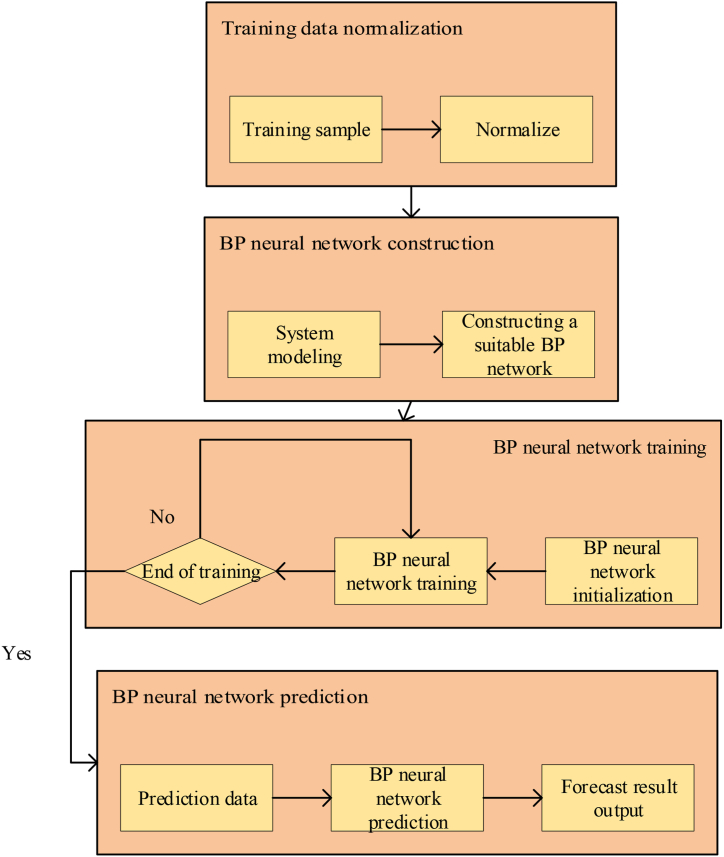
Modeling process of BPNN.

The modeling process of BPNN is shown in Fig. 2.

From Fig. 2, the modeling steps of BPNN are as follows. The first step is to normalize the training data. The second step is to construct the BPNN. The third step is to train the BPNN. The last step is to use the BPNN for prediction after the network is successfully trained, and the forecast result is output [20].

Based on the above content, BPNN has widespread application in the fields of pattern recognition and prediction due to its excellent performance when dealing with complex datasets. Moreover, this model can enhance accuracy and performance by adjusting weights through the backpropagation algorithm. The network structure of BPNN is capable of effectively handling nonlinear relationships, making it suitable for complex tactical analysis and diagnostics. Therefore, this paper chooses BPNN for a more precise analysis and evaluation of the tactical performance in men's singles tennis.

### 3.3 Construction of a tactical diagnostic system for tennis matches

**Fig. 3:**
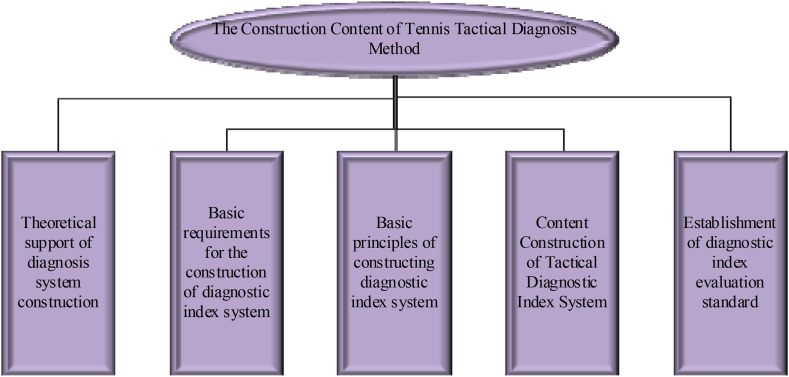
The construction content of the tennis tactical diagnosis method.

This paper studies the tennis training and teaching of college students and builds a tennis tactical diagnosis method based on healthy sports. The specific content is shown in Fig. 3.

From Fig. 3, the construction of the tennis tactics diagnostic method mainly includes the following contents. The first is to construct the theoretical basis of the tennis tactical diagnostic index system. The second is the basic requirements for constructing a tennis tactical diagnostic index system. The third is the basic principle of making the tennis tactical diagnostic index system. The fourth is the empirical construction process of the diagnostic index system. The fifth is formulating evaluation criteria for the diagnostic index system [21].

#### 3.3.1 Theoretical support for the construction of the diagnostic system

##### 3.3.1.1 System science theory

The idea of systems theory was first proposed in the middle of the 20th century. Later, through continuous enrichment and improvement, a set of theories, categories, and methods with their characteristics have been formed. The practical value of systems theory has been gradually valued and accepted by people over time. Besides, it has been continuously applied in science and practice in modern society [22].

A system is an organic whole with specific goals and functions composed of several elements interconnected, interacting with, and transforming into each other. Each whole in the system has its unique function. The number of elements of the system is sufficient. The differences between the elements are large and cannot be integrated according to a particular attribute. However, different parts can be divided according to a specific attribute. Then, several subsystems can be formed. Each subsystem has its structural composition. This structure will show the corresponding function. Structure and function superimpose to create the structure and function of a large system [23].

In summary, systems theory aids students in better comprehending the constituent elements of tennis matches, assisting them in achieving a more comprehensive understanding and analysis of various phases and tactical aspects of the game.

##### 3.3.1.2 Group training

The theory of sports training is derived from sports practice. According to “Training Science”, the theory of item group training starts from the scientificity and practicality of the classification system. Based on the needs of actual tasks, it divides sports into groups according to the dominant factors of athletes' competitive ability, performance evaluation methods, and technical action structures. This theory is constructed based on classifying the dominant factors of athletes' competitive ability [24]. The establishment of sports group training theory helps to explore and reveal the training rules of group sports and scientifically and rationally formulate competitive sports development strategies. It also facilitates the selection and flow of competitive talent [25].

##### 3.3.1.3 Competitive process of the basic unit of the competition group with the racket and the net

Racket and net competitions, such as table tennis, tennis, and badminton, involve athletes from both sides using a racket as a tool, a ball as a medium, and a batting action as the basic means of play. Within the boundaries set by the rules, athletes take turns hitting the ball into the opponent's court area, with the cycle repeating until one side commits a foul or error. The theory of the basic unit competitive process, which involves holding the racket and separating the net, systematically describes the organizational structure, stage characteristics, and multi-beat structure of this process [26].

##### 3.3.1.4 Tennis technical and tactical analysis theory

Many tennis scholars have continued to pay attention to and discuss tennis skills and tactics. For example, self-designed statistical indicators have been pointed out and applied to in-depth diagnostic studies of elite athletes' tactics and tactics [27]. The technical statistical indicators based on the competition's official website are proposed. The rules of the winning factors of tennis match tactics and tactics are discussed and applied to the diagnostic analysis of the tactics and tactics of elite athletes [28]. These representative research results support the scientific training and competition of Chinese tennis athletes and promote the development of Chinese tennis. The tennis technique and tactics diagnostic analysis method proposed by the predecessors has laid a solid theoretical foundation and basis for this paper [29].

The “Group Training” theory introduces a scientific and practical classification method to better understand and analyze student-athletes' competitive abilities. By categorizing students into different groups, this paper can formulate more targeted training strategies based on the dominant factors of their competitive abilities, crucial for enhancing their tactical proficiency. Additionally, the theory of the “Competitive Process of the Basic Unit of the Competition Group with the Racket and the Net” provides an in-depth understanding of fundamental competitive processes, especially in racket and net sports. This theory aids this paper in gaining a more comprehensive grasp of the organizational structure and characteristics of different phases of tennis matches, enabling better training and competitive strategies for students. Lastly, the “Tennis Technical and Tactical Analysis Theory” imparts valuable insights into tennis techniques and tactics, useful for analyzing student-athletes' tactical proficiency. The earlier paper provides a robust theoretical foundation for this study, facilitating a more scientific assessment and enhancement of tactical proficiency in collegiate tennis training. Consequently, these theories offer crucial theoretical support for this paper and hold promise for widespread application in collegiate tennis training, ultimately enhancing the comprehensive competitive abilities of the students.

#### 3.3.2 Basic requirements for the construction of the diagnostic index system

The tennis tactical diagnostic system's basic requirements are as follows: 1) The composition of diagnostic indicators must be complete. 2) The names of diagnostic indicators must have correct meanings and basis. 3) Diagnostic indicators should have precise statistical scales. 4) Reasonable calculation methods should be used for the values of statistical indicators [30].

#### 3.3.3 Basic principles for the construction of the diagnostic index system

**Fig. 4:**
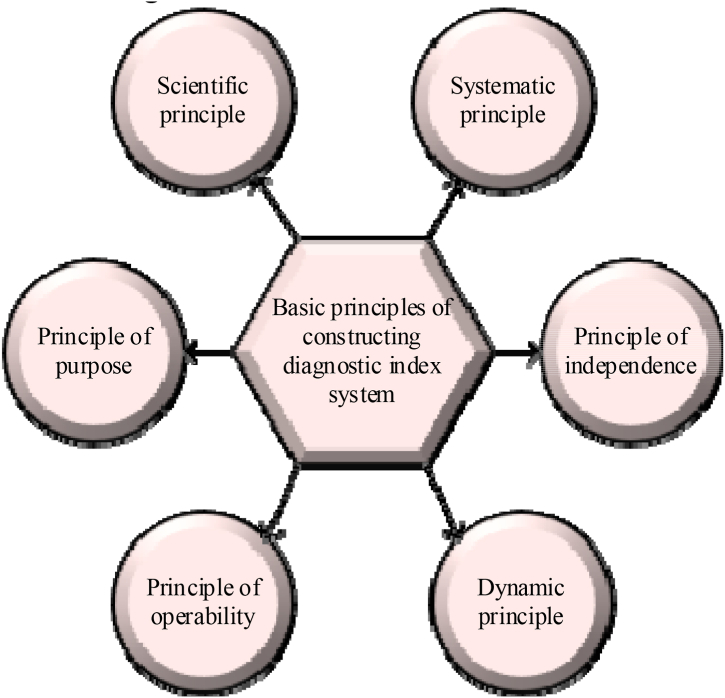
Basic principles for the construction of the diagnostic index system.

The basic principles followed by the tennis tactical diagnostic index system constructed here are shown in Fig. 4.

##### 3.3.3.1 The principle of scientificity

All academic research activities must adhere to the principle of scientificity. This means that the theoretical foundation of the evaluation index system should be comprehensive and logically sound. The selected indicators should scientifically reflect the object of knowledge to accurately diagnose the tactical level of male tennis athletes [31].

##### 3.3.3.2 Systematic principles

When constructing the tennis tactics diagnostic index system, the tennis tactics level should be analyzed as an organic system. It is crucial to use several organically related but mutually independent diagnostic indicators to reflect the current situation of the athletes’ tactical level, rather than simply stacking several indicators. Furthermore, the design of the indicator system should systematically and scientifically portray the overall picture of the tactical level, ensuring the comprehensiveness and credibility of the diagnosis [32].

##### 3.3.3.3 The principle of purpose

The construction of the diagnostic index system serves a diagnostic purpose and provides a basis for judging the diagnostic results. This diagnosis aims to enhance the tennis tactics level of Chinese tennis athletes and offer a reference for the scientific teaching, training, and competition of general tennis courses. Therefore, the selected and designed diagnostic indicators should not only detail each tactical link of the athlete but also capture the overall tactical level macroscopically. The established diagnostic criteria can scientifically reflect the correlation between the tactical level of tennis athletes and their indicators, improving the effectiveness of the diagnostic criteria [33].

##### 3.3.3.4 The principle of independence

The principle of independence should be implemented when decomposing the overall goal into specific diagnostic indicators. Various specific diagnostic indicators should be interdependent and interrelated to form an organic comprehensive target system. Additionally, indicators should be independent of each other, and the diagnosis and assessment of each specific indicator should be carried out independently. The overall design of the indicator system cannot lack any one indicator to avoid preliminary diagnosis. Moreover, indicators that cover each other cannot appear, preventing imprecise overlap in diagnosis [34].

##### 3.3.3.5 The principle of operability

The value of the diagnostic index system can only be realized when put into practice. This necessitates the high operability of the diagnostic index system. The ability to collect data timely and effectively is the basis for diagnosis. On one hand, it is necessary to fully reflect the connotation of the athlete's tactical level to make it targeted. Also, the current situation of the athlete's tactical level can be revealed, and suggestions for improvement can be proposed. On the other hand, there should not be too many diagnostic indicators; they should be simplified and easy to understand, making data readily available for analysis, diagnosis, and monitoring. Additionally, the correlation between indicators should be reduced as much as possible to avoid duplication and crossover [35].

##### 3.3.3.6 Dynamic principle

The dynamic nature of diagnostic indicators refers to the design process of modifying, adding, deleting, supplementing, and perfecting the diagnostic indicators according to the development and changes in scientific development theory and tennis practice. This adjustment is necessary to accommodate differences in all aspects of the athletes’ competitive ability. Furthermore, it is crucial to continuously develop theories, diagnostic theories, and diagnostic index systems to keep pace with the times and improve continuously [36].

#### 3.3.4 Content construction of tactical diagnostic index system

**Fig. 5:**
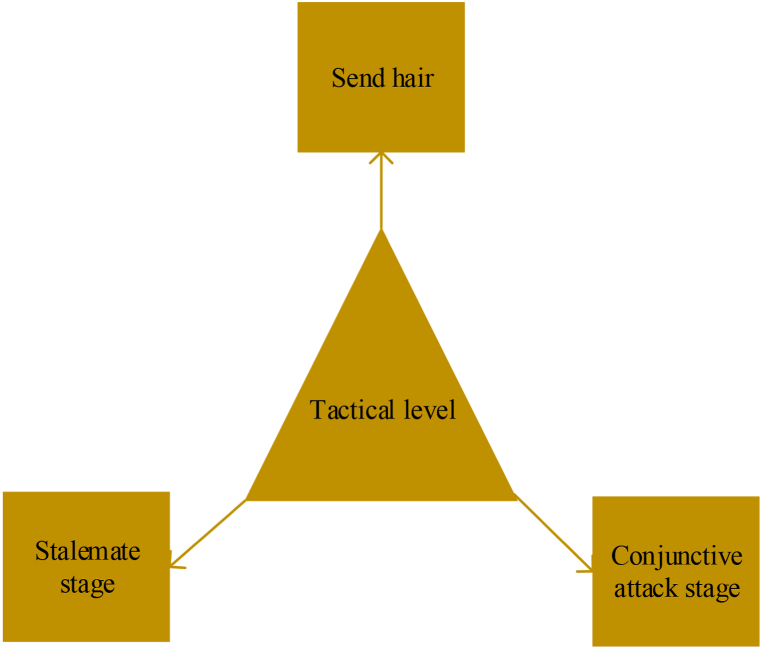
Schematic diagram of the initially determined tactical diagnostic indicators for men's tennis matches.

In the analysis of the characteristics of tactics employed in men's singles competition on hard court tennis, the relevant literature is consulted. Additionally, insights from experts regarding the viability of diagnosing the overall competitive ability of men's tennis singles in stages are examined. The experts contribute valuable suggestions and opinions to the discussion. Consequently, this paper categorizes the overall tactical level of men's singles tennis into distinct stages: the sending and receiving stage, conjunctive attack stage, and stalemate stage, as illustrated in Fig. 5.

Then, the overall tactical level of men's singles tennis competition is further divided into three stages and six links. Table 1 shows the specific content of the established indicators.

**Table 1:** Contents of tennis tactics diagnostic index system.

Contents of first-level indicators	Contents of secondary indicators	Contents of the third-level indicators
Tennis tactical ability	Sending and receiving stage	Serving stage
		Sending and receiving stage
	Conjunctive attack stage	Serving and catching stage
		Receiving and catching
	Stalemate stage	Stalemate A link
		Stalemate B link

Moreover, 12 sports-related experts and coaches are surveyed again to determine whether the constructed diagnostic indicators are feasible. Table 2 reveals the results.

**Table 2:** Expert survey results of “Tennis tactical diagnostic indicators”.

Evaluation indicators	Recognize very much	Recognize	Remain neutral	Less recognized	Not recognized
Proportion of people/%	83	17	0	0	0

According to Table 2, 83% of the experts express a high degree of recognition for the diagnostic index system, while 17% acknowledge it. Based on these findings, the assessment of an athlete's overall tactical level can be conducted by evaluating the performance levels across six tactical components: the serving link, receiving and serving linking, serving and catching link linking, receiving and catching link linking, stalemate I link, and stalemate II link.

#### 3.3.5 Development of diagnostic index evaluation criteria

The paper mentioned above draws upon the principles of sports for health and systems theory. In conjunction with existing research and expert recommendations, it has revised the initial classification and assessment criteria. Simultaneously, in alignment with the principles of scientific rigor, practicality, and relevance to the actual conditions of tennis matches, a set of assessment criteria has been developed for evaluating tennis tactical proficiency. These criteria are intentionally interconnected with the tennis tactical diagnostic indicator system presented in this paper, aiming to provide a more comprehensive understanding and enhancement of tactical proficiency in collegiate tennis training.

The adjustment of the preliminary classification evaluation criteria is informed by interviews with twelve experts, drawing on their experiences, suggestions, and the research findings of predecessors. This paper refines the evaluation criteria for the tennis tactical level, guided by the principles of science, ease of operation, and the ability to reflect the actual situation of tennis competition. The formulated evaluation criteria are detailed in Table 3 [37].

**Table 3:** Tennis tactical level evaluation criteria.

Index name/evaluation criteria	Poor	General	Good	Excellent
Serving link	Below 85	85∼90	90∼96	96 and above
Serving and catching link-linking	Below 47	47∼56	56∼66	66 and above
Stalemate A link	Below 35	35∼46	46∼57	57 and above
Receiving and serving linking	Below 24	24∼32	32∼41	41 and above
Receiving and catching link-linking	Below 28	28∼37	37∼47	47 and above
Stalemate B link	Below 32	32∼43	43∼55	55 and above

### 3.4 Design of tennis tactics diagnosis model based on BPNN

**Fig. 6:**
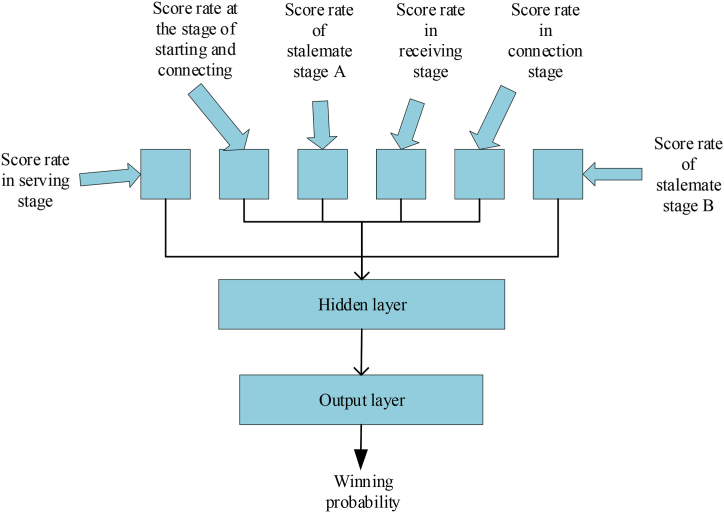
Structure diagram of NN model for men's tennis singles match.

The structure of the tennis tactical diagnostic model based on BPNN constructed here is demonstrated in Fig. 6.

Fig. 6 suggests that the tennis tactical diagnostic model developed in this study follows the three-layer topology characteristic of the classic BPNN. The input layer comprises six nodes, representing the scoring rate of the six tactical links. The output layer consists of one node, indicating the probability of winning the game. Through a trial-and-error approach, the number of hidden layer nodes in the prediction model is determined to be ten. The training function from the input layer to the hidden layer is the nonlinear S-function Tansig, while the transfer function from the hidden layer to the output layer adopts the linear function Pruelin.

## 4 Experimental Design and performance evaluation

### 4.1 Datasets collection

#### 4.1.1 Case design of tennis tactical diagnostic index system

The immediate assessment of the tennis tactics employed by Chinese tennis athletes Zhang Ze and Wu Di in international tennis competitions serves as the focal point for studying the application of the tennis tactical diagnostic index system.

#### 4.1.2 Experimental design of tennis tactics diagnostic model based on BPNN

A total of 120 men's tennis hard-court singles matches were collected and utilized as input for training samples. Among these, 80% were randomly assigned to the training set, 10% for the validation set, and 10% for the test set. All data were sourced from internet searches.

To meet the experimental requirements, this paper performed data preprocessing. This involved several steps, including removing duplicates and outliers through data cleaning, standardizing data to ensure consistency across different scales, conducting feature engineering to select and create relevant features, segmenting data into training, validation, and test sets, digitizing categorical data through data encoding, balancing imbalanced datasets, and utilizing data visualization to enhance understanding during the data processing phase. These measures are crucial to ensuring data quality and reliability, thereby providing a solid foundation for further research.

### 4.2 Experimental environment

The tennis tactical diagnosis model based on BPNN is constructed by the default Levenberg-Marquardt algorithm in the MatLab toolbox.

### 4.3 Parameters setting

The performance of the model heavily relies on parameter settings; therefore, the parameters are configured to effectively capture the features of tennis tactics. The number of input layer nodes in the model is set to 6, corresponding to six tactical indicators. Meanwhile, the number of output layer nodes is 1, representing the prediction of the match win rate. Through experimentation and adjustment, the number of hidden layer nodes is determined to be 10.

This paper employs Tansig as the training function and Pruelin as the transfer function to ensure that the model possesses high accuracy and generalization capability. To balance training speed and accuracy, the learning rate is set to 0.02. In contrast, the convergence threshold is set to 0.001 to prevent the model from continuing training once it has achieved sufficient accuracy, thus conserving computational resources.

These parameter settings are carefully considered to guarantee that the model can successfully perform tactical diagnostics and provide meaningful results regarding the players’ tactical proficiency.

Table 4 shows the specific settings of the parameters for the tennis tactical diagnosis model based on BPNN designed here.

**Table 4:** Parameter settings.

Parameter name	Value
The number of nodes in the input layer	6
The number of nodes in the output layer	1
The number of nodes in the hidden layer	10
Training function	Tansig
Transfer function	Pruelin
Learning rate	0.02
Convergence accuracy	0.001

Furthermore, the experimental metrics utilized in this paper primarily consist of the Mean Square Error (MSE) and the utility value. The utility value serves as an indicator to assess the tactical performance of tennis players in various match phases, such as serving and receiving phases, collaborative offensive phase, and deadlock phase. This value typically reflects the quality and effectiveness of a tennis player's tactical performance in specific match phases.

### 4.4 Performance evaluation

#### 4.4.1 BP model training results

**Fig. 7:**
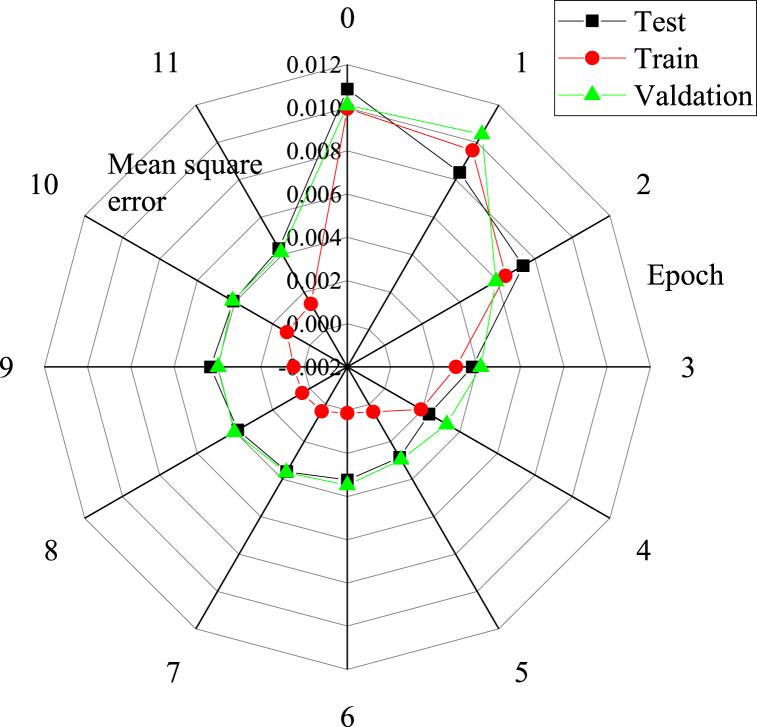
The training error curve of the prediction model for men's tennis singles match.

The BP model is constructed using the MatLab platform, and the training error results of the BP model are revealed in Fig. 7.

In Fig. 7, it is evident that the prediction model for men's tennis singles matches successfully converged after 11 learning iterations. The best mean squared error on the validation set is recorded at 0.00037146, while the mean squared error for the training set is 0.0104. These results indicate that the network training speed for the men's tennis singles match prediction model is rapid, and the training effect is highly favorable.

#### 4.4.2 Application effect of tennis tactical diagnostic system

**Fig. 8:**
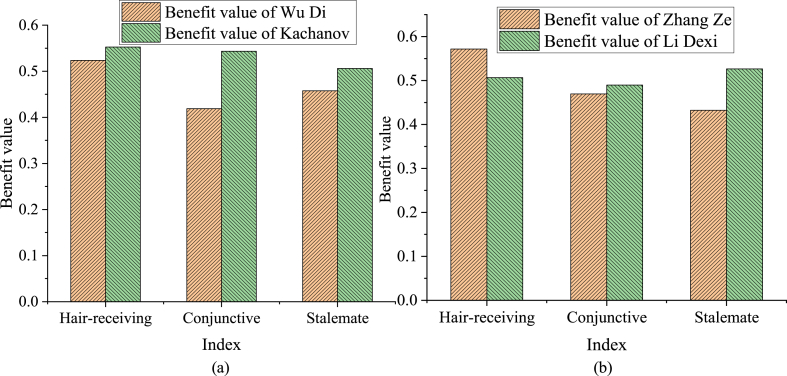
Instant diagnosis results of Wu Di and Zhang Ze's tennis tactical level (a) The comparison chart of the three-stage benefit value in the competition between Wu Di and Chanov; (b) the comparison chart of the three-stage benefit value in the competition between Zhang Ze and Li Dexi.

Wu Di and Zhang Ze's tennis tactical level is instantly diagnosed using the tennis tactical diagnosis system constructed here, and the results are shown in Fig. 8(a) and (b).

From Fig. 8(a) and (b), Wu Di's three-stage tactics are not as effective as his opponents in Wu Di's match with Khachanov. Especially in the conjunctive attack stage, the scoring rate is assessed as failing, and the benefit value is far lower than the opponent. In addition, Zhang Ze's advantage lies in the sending and receiving stage in the match between Zhang Ze and Li Dexi. The benefit value is 0.5716, which is significantly higher than Li Dexi. In the conjunctive attack stage, Zhang Ze's benefit value is slightly lower than Li Dexi's. Moreover, Zhang Ze's obvious weak link is in the stalemate stage. The benefit value is 0.4322, much lower than Li Dexi's 0.5264.

### 4.5 Discussion

As proposed by Hidayat et al. [38], the temporal and spatial application of individual techniques and combination tactics does not fully capture the specific details of athletes' tactical usage in matches [38]. Therefore, this paper integrates BPNN technology with the practical context of tennis education to develop a relatively comprehensive tennis tactical assessment system.

In previous research on tennis teaching and training, scholars primarily analyzed tennis skills and tactics using index data from the official event website for descriptive statistical research. While these indicators enhance fans' viewing experience, they are macro and general, lacking specificity in the use and effects of specific techniques and tactics. For instance, the time and space utilization of single technical tactics and combined technical tactics fail to reflect the specific information of athletes' tactical use in the game.

In the context of medical and physical education, this paper illustrates the connection between medical care and physical education. Additionally, it constructs a relatively complete tennis tactics evaluation system using NN technology combined with the current state of tennis teaching. During the research process, the BPNN demonstrates a positive effect on training outcomes and prediction accuracy.

In conclusion, the system effectively identifies the strengths, weaknesses, and existing problems in athletes' tactical use during games. After constructing a diagnostic index system and evaluation criteria, post-match diagnosis and evaluation of Zhang Ze and Wu Di were conducted. This diagnostic index system allows for instant diagnosis and analysis of men's tennis hard court singles match tactics during and after the game, providing a detailed understanding of athletes' tactical use and their states. It serves as a foundation for formulating tactical strategies and training arrangements for athletes during and after games, facilitating specific training and competition guidance.

## 5 Conclusion

### 5.1 Research contribution

The research on the diagnostic index system and comprehensive evaluation method of tennis tactics represents an emerging field in tennis. This paper systematically explores the entire process of constructing, diagnosing, and thoroughly evaluating the tennis tactical diagnostic index system. The main conclusions drawn from this research process are summarized as follows:

(1) A tactical diagnostic index system for men's tennis hard singles matches is established based on expert experience and the research of previous scholars. This system comprises three secondary indicators and six tertiary indicators. Subsequently, an evaluation standard is defined, enabling comprehensive and detailed diagnosis and analysis of athletes' tactical strength from the macro to the micro level.
(2) Diagnostic indicators at all levels are utilized for real-time diagnosis and research of athletes during and after the game. This analysis enables a precise examination of athletes' tactical use in a specific match, providing insights into their state. Moreover, it serves as a foundation for formulating athletes' tactical strategies and training arrangements during and after the game, facilitating targeted training and competition guidance.

The construction of the tennis tactical diagnostic index system, along with the establishment of evaluation criteria and the use of diagnostic indicators, allows for the diagnosis and evaluation of athletes' tactical strength through various methods. This not only offers ideas and methods for the quantitative analysis of tactical performance in men's singles tennis but also provides theoretical references for optimizing general tennis course training and teaching for college students.

The experimental results presented herein provide a quantitative approach and methodology for analyzing the tactical performance of men's singles tennis. Furthermore, this paper contributes theoretical insights to the training and teaching of undergraduate tennis courses, offering valuable input for enhancing athletes' tactical proficiency, nurturing the next generation of talented tennis players, and advancing tennis education. These findings have the potential to furnish coaches and athletes in the tennis field with more useful information, ultimately contributing to improved competitive performance and career advancement.

### 5.2 Future works and research limitations

The shortcomings are:

The tactical diagnostic index system for tennis primarily focuses on men's singles competitions on hard courts, excluding involvement in clay courts, grass courts, carpet courts, and women's events. Building upon the aforementioned scope, future research endeavors should aim to enhance the tactical diagnostic index system, making it applicable to clay and grass games. Additionally, there is a need to direct attention towards developing diagnostic methods tailored to the tactics of female athletes.

The ongoing refinement of the tactical diagnostic index system is essential to align with the dynamic landscape of tennis tactics and the evolving reforms in game rules. Continuous efforts should be made to enrich and improve the constructed system through practical applications. This iterative process will ensure the relevance and effectiveness of the system in keeping pace with the developments in tennis tactics and the ever-changing landscape of the sport.

## CRediT authorship contribution statement

**Yu Zhang:** Writing – original draft, Visualization, Validation, Software, Methodology, Formal analysis, Data curation, Conceptualization.

## Declaration of competing interest

The authors declare that they have no known competing financial interests or personal relationships that could have appeared to influence the work reported in this paper.
